# A Rare Case of Pulmonic and Aortic Valve Infective Endocarditis: A Case Report

**DOI:** 10.7759/cureus.31820

**Published:** 2022-11-23

**Authors:** Miguel E Perez-Viloria, Kalei Lopez, Fayeza Malik, Olga Lopez, Puja Yatham, Rayik Malik, Gerald Rosen

**Affiliations:** 1 Department of Anesthesiology, Mount Sinai Medical Center, Miami Beach, USA; 2 Department of Anesthesiology, Florida International University, Herbert Wertheim College of Medicine, Miami, USA; 3 Department of Psychiatry, Florida International University, Herbert Wertheim College of Medicine, Miami, USA; 4 College of Medicine, Florida International University, Herbert Wertheim College of Medicine, Miami, USA; 5 College of Medicine, University of Miami, Miami, USA

**Keywords:** valve replacement, aortic valve, pulmonic valve, infective endocarditis, case report

## Abstract

Infective endocarditis (IE) is a microbial infection affecting cardiac valves. IE most often affects the aortic valve and is commonly caused by community-acquired, penicillin-sensitive streptococcus that enters through the oral cavity. In this report, we present a case of a 66-year-old man with a medical history of congenital pulmonic stenosis status after pulmonic valve (PV) repair. The patient underwent a transesophageal echocardiogram showing a 1 cm × 0.7 cm mobile vegetation attached to the ventricular aspect of the right coronary aortic cusp and a 1.1 cm × 0.5 cm mobile vegetation attached to the arterial aspect of the PV cusp. In conclusion, concomitant right- and left-sided IE is an exceedingly rare condition. Due to its rarity and complexity of presentation, pulmonic valve endocarditis (PVE) requires a multidisciplinary approach to its perioperative management to prevent systemic complications.

## Introduction

Infective endocarditis (IE) is a microbial infection of the endocardial surface of native valves, prosthetic valves, or implanted cardiac devices [1]. The incidence of IE is thought to be rare with between 3 and 10 cases per 100,000 per year and is most often found to affect the aortic valve (AV) followed by the mitral valve (MV), with AV endocarditis (AVE) involvement accounting for over 50% of total IE admissions [1-3]. Throughout most parts of the world, the primary risk factor for IE is rheumatic heart disease, which accounts for up to 66% of all reported cases [4,5]. In developed countries, IE results from bacterial or fungal infections of the endocardial surface, and risk factors include the presence of a prosthetic heart valve, structural or congenital heart disease (CHD), intravenous drug use (IVDU), and recent history of invasive procedures [6]. Right-sided endocarditis is rare, accounting for only 5%-10% of all cases of IE, with many of these cases involving the tricuspid valve [1]. This discrepancy in incidence can largely be attributed to several factors, such as the low prevalence of conditions that typically would affect the right-sided heart valves, vascularity differences between the right-sided and left-sided endothelium, lower pressure gradient across the right-sided valves, and lower blood oxygen content within the right side of the heart [7-9]. Further, the presentation of right-sided endocarditis can differ significantly from that involving the left chambers of the heart. Pulmonary involvement is the most striking difference, in that it is involved in 80% of right-sided IE cases and can manifest as atelectasis or cavitation within the lower lobes [3]. Pulmonic valve endocarditis (PVE) is the least common, accounting for 1.5%-2.0% of total IE admissions, and may be missed if patients do not have the typical risk factors or features of right-sided endocarditis [9]. While the etiology of AVE is usually attributed to oral ingestion of community-acquired, penicillin-sensitive streptococcus affecting the young adult populations, PVE is usually due to IVDU [10]. Other contributors to right-sided endocarditis generally include central venous catheter placement, alcoholism, dental extraction, and CHD [9,11,12].

Diagnosis of PVE is based on a combination of clinical findings such as persistent fever, pulmonary symptoms such as cough and dyspnea, pulmonic regurgitant murmur, and predisposing conditions such as IVDU or history of valve abnormalities [6]. The modified Duke criteria can further help identify the diagnostic likelihood of IE as either definite or possible using the major, minor, and pathological criteria [1,13-15]. Diagnostic categorization of definite IE is present with any of the following: ≥2 major criteria, ≥1 major criterion + ≥3 minor criteria, ≥5 minor criteria, or ≥ pathologic criterion [1,15]. Possible IE is satisfied with any of the following: ≥1 major criterion + ≥1 minor criterion or ≥3 minor criteria [1,15]. While the most common causative pathogens involved in PVE include *Staphylococcus aureus*, coagulase-negative staphylococci, and group B streptococci, treatment of IE can vary based on the severity of the infection and characteristics of involved structures [3]. Empiric antibiotics are generally indicated for hemodynamically unstable patients or hemodynamically stable patients with acute symptoms and/or complications [8]. Targeted antibiotics are recommended for all patients and can be initiated immediately in stable patients with no complications after culture results are available [1,8]. Surgical intervention is typically reserved for cases with persistent bacteremia and/or complications such as recurrent septic pulmonary embolism or vegetation >10 mm [16-18].

This case report highlights the importance of early recognition and careful management of concomitant right-sided and left-sided IE in the perioperative setting, with specific emphasis on PVE, given the limited number of reports in the literature regarding this pathology. To our knowledge, there have only been two cases ever reported regarding concomitant right- and left-sided IE, with no case reports of concomitant right- and left-sided IE involving the pulmonic valve (PV) [19]. Overall, this condition warrants early management and frequent follow-up to both prevent long-term systemic complications and reduce the risk of mortality. 

## Case presentation

**Figure 1:**
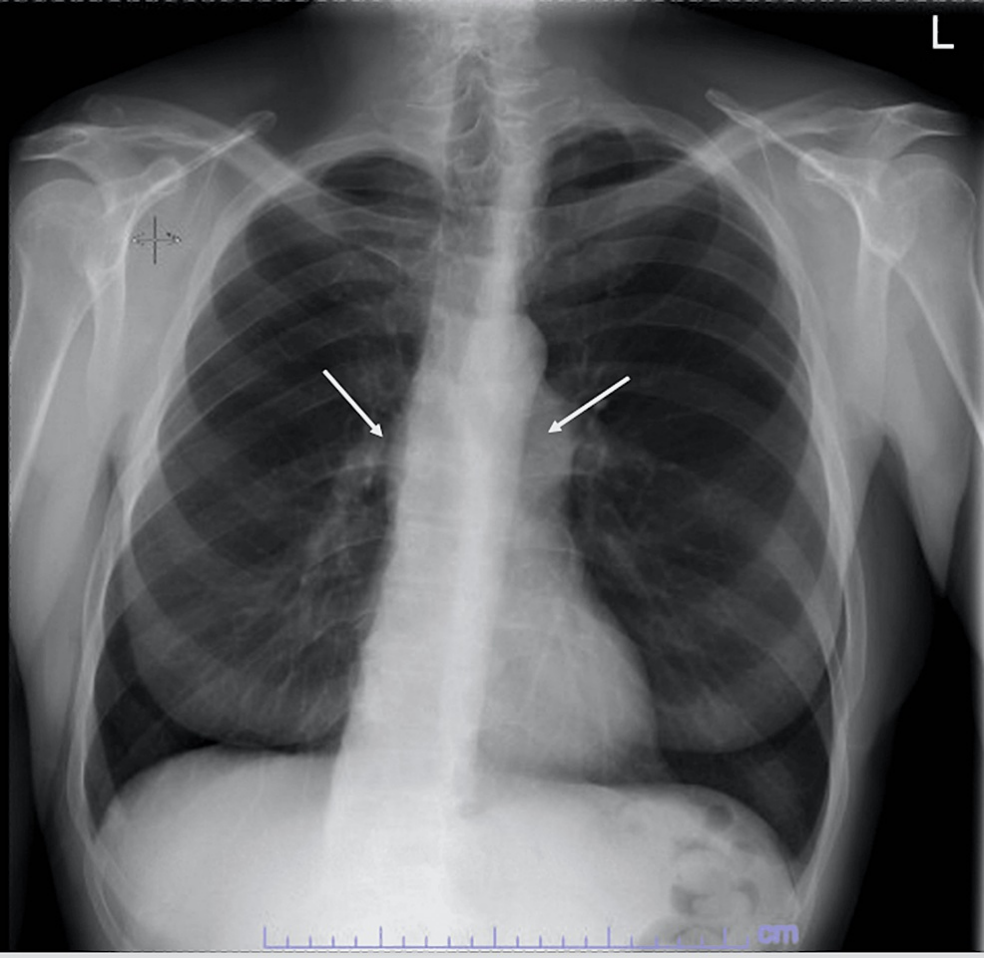
X-ray suggestive of mild enlargement of the proximal pulmonary arteries.

A 66-year-old man with a medical history of congenital pulmonic stenosis status post-PV repair via sternotomy at 5 years of age, moderate aortic insufficiency, May-Thurner syndrome, and hypertension was admitted for a five-month history of cough, one-month history of weight loss, nighttime chills, and orthopnea one week following a dental cleaning without antibiotic prophylaxis. One month before admission, the patient’s cough was evaluated in an outpatient setting, and he was found to have pulmonary function tests within normal limits, an X-ray suggestive of hyperinflation and mild enlargement of the proximal pulmonary arteries but otherwise noncorrelative (Figure 1), and to be nonresponsive to oral or inhaled steroids. Three weeks into experiencing increasing weight loss and nighttime chills, he was evaluated by an infectious disease doctor in an outpatient setting, who suggested that he may have IE.

**Figure 2:**
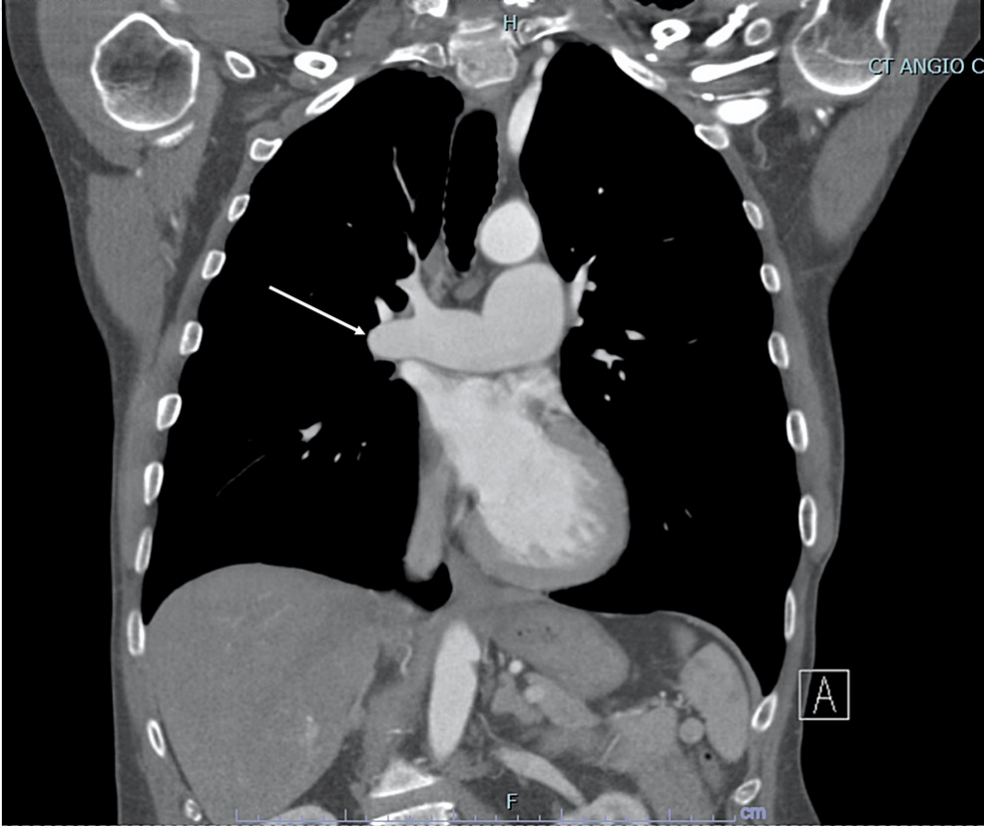
CTA chest identifying marked enlargement of the main central pulmonary arteries. CTA, computed tomography angiography

**Figure 3:**
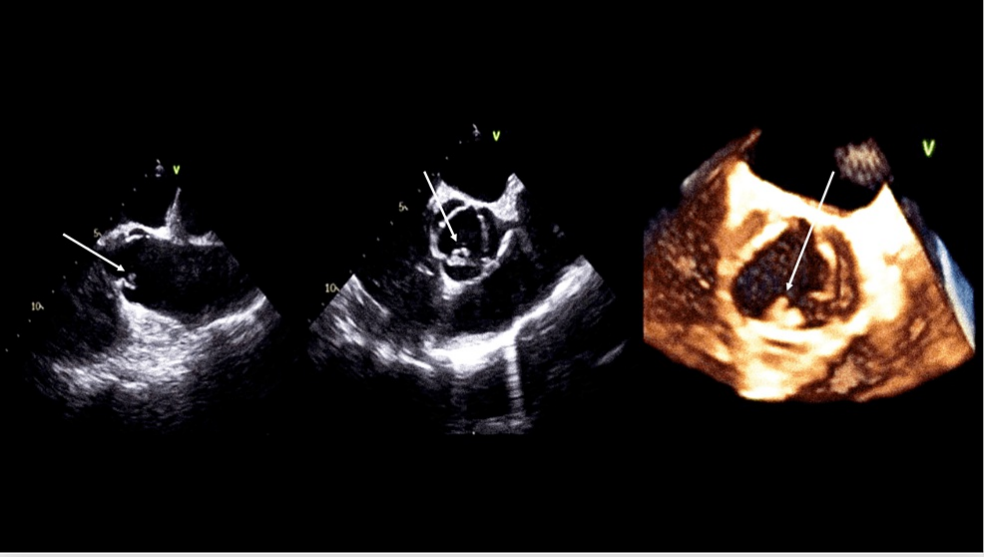
TEE images showing vegetation in the AV. AV, aortic valve; TEE, transesophageal echocardiography

**Figure 4:**
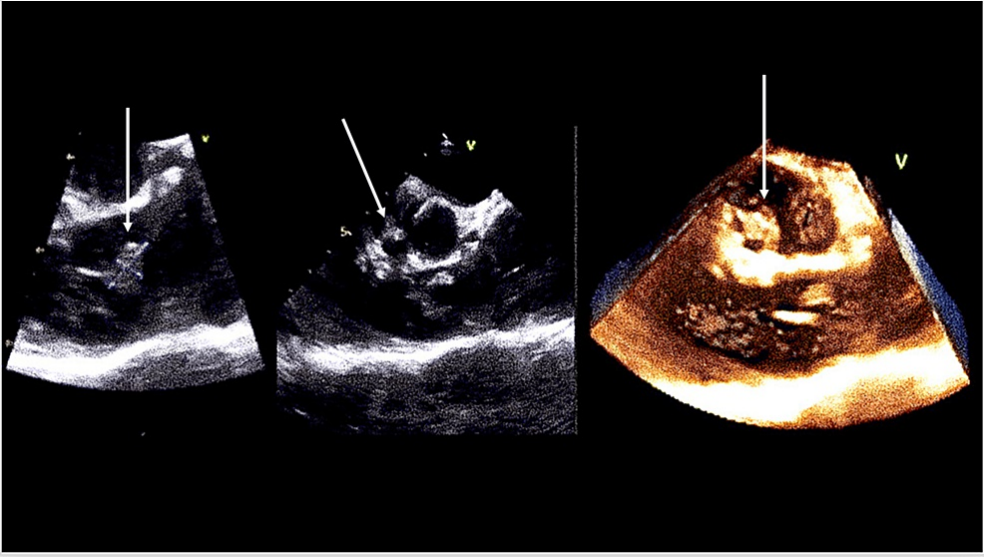
TEE images showing vegetation in the PV. TEE, transesophageal echocardiography; PV, pulmonic valve

**Figure 5:**
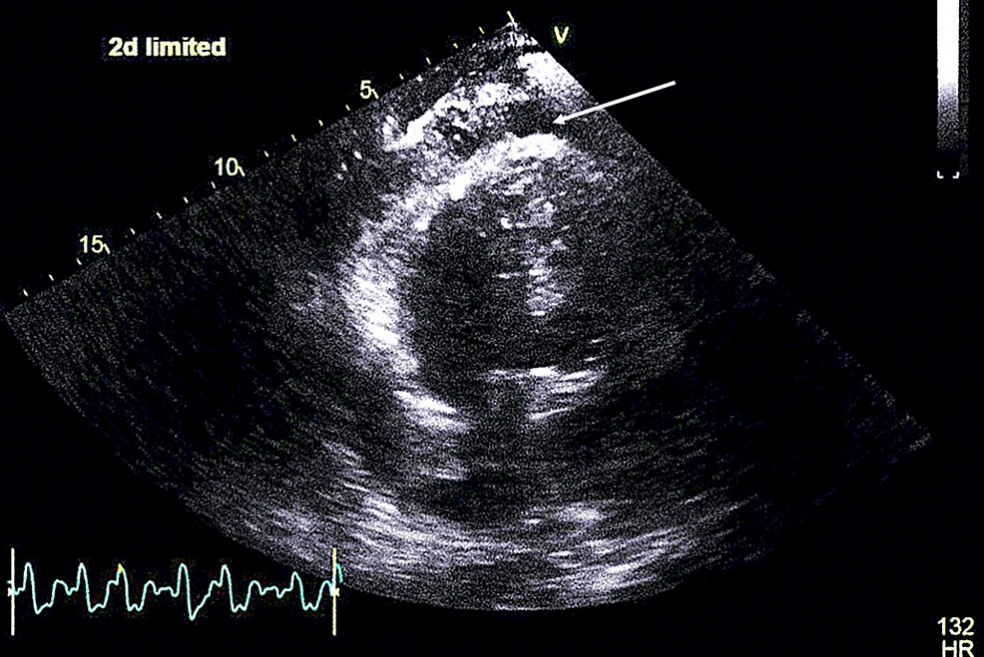
TEE showing moderate pericardial effusion. TEE, transesophageal echocardiography

On admission, his vital signs were significant for an elevated BP 131/66 mmHg, elevated HR 104 bpm, and elevated RR 18 bpm. A workup was done including labs (Table 1) and a positive culture on Admission Day 1 growing gram-positive cocci in pairs and chains (*Streptococcus mitis*). Admission labs were also significant for an elevated C-reactive protein (CRP) and erythrocyte sedimentation rate (ESR). Further imaging was evaluated for guidance in plan management. CT chest without contrast found no focal consolidations to suggest pneumonia but did find a marked enlargement of the main central and left pulmonary arteries consistent with pulmonary valve stenosis and other noncontributory findings (Figure 2). Transthoracic echocardiogram (TTE) was limited, in that besides the findings of moderate-to-severe aortic regurgitation (3+) and mild PV regurgitation (1+), it did not show anything else relevant. A transesophageal echocardiogram (TEE) was the next recommended step for better visualization of suspected right-sided pulmonic endocarditis. On Admission Day 2, the patient underwent a TEE procedure that showed a 1 cm × 0.7 cm mobile vegetation attached to the ventricular aspect of the right coronary aortic cusp (Figure 3) and a 1.1 cm × 0.5 cm mobile vegetation attached to the arterial aspect of the PV cusp (Figure 4). The patient was started on IV vancomycin at presentation and then continued with the addition of IV ceftriaxone on Admission Day 2. After a multidisciplinary discussion of the case, surgery was recommended, and on Admission Day 6, the patient underwent uncomplicated reoperative sternotomy, aortic valve replacement (27 mm Carpentier-Edwards Magna-Ease pericardial tissue valve, Edwards Lifesciences, Irvine, CA, USA), and pulmonary valve replacement (27 mm cadaveric homograft). After being extubated, the patient developed a moderate pericardial effusion in the postanesthesia care unit (Figure 5), with echocardiographic signs of early cardiac tamponade requiring an urgent subxiphoid pericardial window. The patient was transferred to a cardiac step-down unit on post-op Day 7. The mediastinal chest tube was removed on post-op Day 9, and the patient was discharged on post-op Day 10 to inpatient rehabilitation in stable condition. 

**Table 1:** Laboratory values. AST, aspartate aminotransferase; WBC, white blood cell; U, unit

Variable	Result	Reference value
WBC count	12.31	4.8 × 10^3^ to 10.8 × 10^3^ microL^–^^1^
Hemoglobin	13.9	14.0-18.0 g/dL
Hematocrit	41.2	42%-52%
Neutrophils relative percentage	85.0	42%-74%
Lymphocytes	6.1	16.0%-45.0%
AST	40.0	15.0-37.0 U/L
Albumin	2.9	3.4-5.0 g/dL

## Discussion

There exists one study describing concomitant right- and left-sided IE, although, notably, without the involvement of the pulmonic valve [19]. The case receiving focus in this study serves as the first description of concomitant right- and left-sided IE with the additional involvement of the PV. Representing only 1.5%-2.0% of total cases of ÌE, PVE has a low incidence, which likely contributes to the literature on PVE being limited to just a few small case series and case reports [20-24]. The scarcity of PVE cases may be due to a range of factors, including the previously mentioned factors of the lower oxygen content of venous blood and lower pressures within the right heart, differences in the endothelial lining and vascularization of the valve, as well as lower incidence of PV congenital malformations or acquired valvular abnormalities [7-9]. Although the most common causes of PVE differ between adults and children, in that congenital malformations more commonly lead to PVE in children and IVDU leads to PVE in adults, several studies have shown that PVE can also occur in patients who underwent orthotopic liver transplantation and chronic hemodialysis [3,25,26].

This study describes a patient who presented with a five-month history of cough, one-month history of weight loss, and diaphoresis after a dental cleaning where he was not given any prophylactic antibiotics. Interestingly, the patient presented without a fever, which is one of the minor criteria for IE, while his elevated RR, BP, and HR indicated a physiologic disturbance taking place. Although this patient lacked a history of IVDU, his recent dental procedure without antibiotic prophylaxis was suspected to be the primary contributing factor to his development of IE, given his pulmonic valve repair at the age of 5 years. It was expected that the repaired valve was susceptible to nidus formation, thus leading to the patient’s development of IE. Aortic insufficiency could be the main contributor to left-sided nidus formation, given the significant AV regurgitation noted on TTE. Further, considering that tricuspid involvement is typically encountered with right-sided endocarditis, the involvement of the PV is indicative of the central role that valvular abnormalities play in the development of IE.

The first line of treatment for PVE caused by viridans streptococci normally involves the use of penicillin or ceftriaxone in combination with an aminoglycoside for at least two weeks. Our patient’s ultimate need for surgical intervention was influenced by a TEE performed, showing two vegetations on each valve measuring 10 mm or greater [15]. TEE is quite useful in the diagnosis of AV and MV IE, with a sensitivity of 85% and specificity of about 90%; therefore, the imaging findings provide adequate support for the diagnosis [27]. In this case report, the patient met many of the modified Duke’s criteria for infectious endocarditis (Table 2) [1,13-15].

**Table 2:** Modified Duke criteria components. HACEK, Hemophilus, Aggregatibacter, Cardiobacterium, Eikenella, Kingella; IgG, immunoglobulin G; IE, infectious endocarditis; IVDU, intravenous drug use

Modified Duke Criteria [1,13-15]	
Criteria	Findings
Major	Two separate blood cultures that are positive for typical organisms; viridans streptococci; Streptococcus aureus; Streptococcus gallolyticus; HACEK group; community-acquired enterococci
	Persistently positive blood cultures with endocarditis-consistent microorganisms; ≥2 positive blood cultures taken >12 hours apart; all 3 or majority of ≥4 separate blood cultures
	One positive blood culture for Coxiella or a titer of antiphase 1 IgG ≥1:800
	Characteristic echogram findings of IE; valvular vegetation; abscess; new valvular regurgitation; prosthetic valve dehiscence
	New valvular regurgitation
Minor	Predisposing conditions; IVDU; prior IE; valvular abnormalities
	Fever >100.4 °F
	Vascular abnormalities; septic infarctions; mycotic aneurysms; Janeway lesions; intracranial hemorrhages
	Immunologic processes; glomerulonephritis; Roth spots; Osler nodes
	Positive blood cultures that do not fulfill major criteria or serologic evidence of infection with typical microorganisms
Pathological	Tissue culture or histology demonstrating microorganisms
	Active endocarditis demonstrated on histology

Surgery is indicated in patients when there is persistent bacteremia despite appropriate antimicrobial therapy, valve destruction, valve incompetence, recurrent pulmonary emboli, or the formation of abscesses [16]. Some of the surgical options include excision of the vegetation with preservation of the valve, debridement of the infected area, or valve repair with autologous or homologous pericardium [16,28,29]. When necessary, pulmonary homografts and stentless xenografts are the most commonly used for the replacement of the valve [30,31]. Right-sided endocarditis has a better prognosis than left-sided endocarditis, as it is more likely to respond to medical therapy [32].

**Figure 6:**
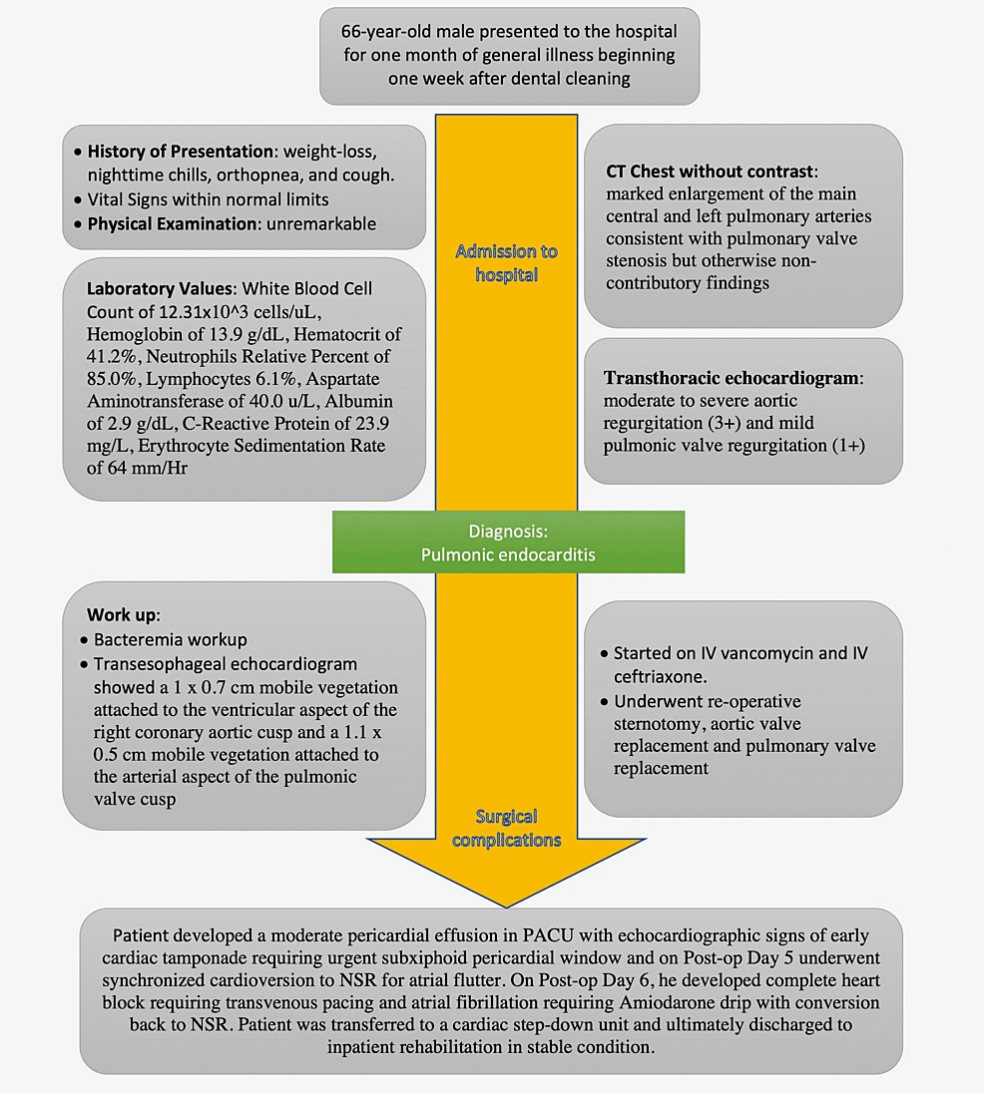
Timeline highlighting the case events. Figure credit: Kalei Lopez CT, computed tomography; IV, intravenous; NSR, normal sinus rhythm; PACU, postanesthesia care unit

A timeline highlighting the case events is shown in Figure 6. The case report presented is in accordance with the Case Report (CARE) guidelines (Appendix).

## Conclusions

In conclusion, PVE is an extremely rare condition. Due to its rarity and complexity of presentation, PVE and concomitant AVE require a multidisciplinary approach to the perioperative management of this disease to prevent systemic complications. Early diagnosis and evaluation of this condition are essential and can be advanced with the use of effective diagnostic criteria and imagining. This report will serve as a contribution to the sparse literature regarding this topic and inevitably contribute to the general understanding of the rare occurrence of concomitant AVE and PVE.

## Figures and Tables

**Figure 7:**
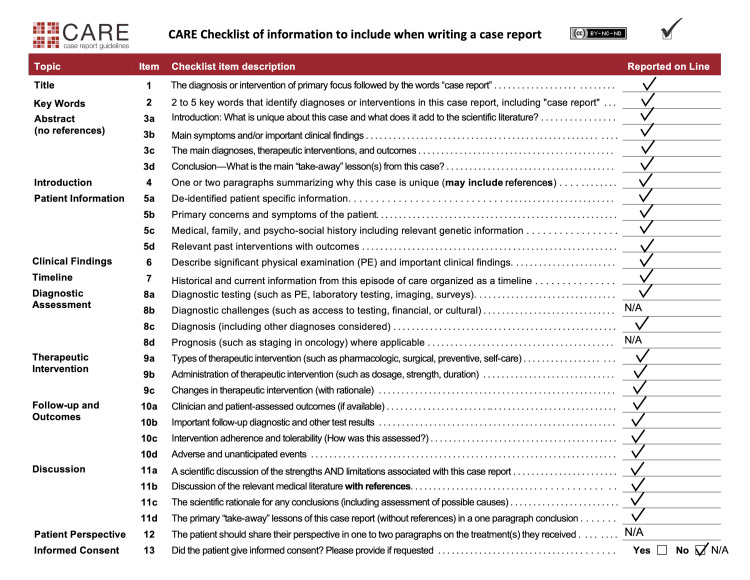
CARE checklist. CARE, Case Report
